# EHMTI-0349. Levetiracetam in the treatment of pediatric headache

**DOI:** 10.1186/1129-2377-15-S1-M9

**Published:** 2014-09-18

**Authors:** T Rizk

**Affiliations:** 1 Habib Medical Group, Al-Takhassusi Hospital, Riyadh, Saudi Arabia

## Introduction

Migraine headache is common among the pediatric and adolescent population, with prevalence rates ranging from approximately

3% to 4% in early ages to as high as 8% to 23% during adolescence. Before puberty, boys have more headaches than girls, but after puberty, migraine headaches occur more frequently in girls. Recent studies have highlighted the potential use of levetiracetam in pediatric migraine prophylaxis, it resulted in significant decreases in mean headache frequency were observed at dosages similar to those used in epilepsy patients, with good tolerability.

## Aims

The study was conducted to evaluate the efficacy and safety of levetiracetam in migraine prophylaxis in young patients.

## Methods

Ten consecutive patients aged 12–21 years with typical or atypical migraine were reviewed prior to and following the administration of levetiracetam for 6 months (750–2250 mg/day).

## Results

Headache score, duration, and frequency showed considerable improvement in all patients compared to baseline. Headaches were eliminated in 3 patients. Electroencephalogram showed dysrhythmia grade 2 or 3 at baseline, which improved to grade 1 in all patients following the treatment period.

## Conclusion

In this pilot study, levetiracetam was effective and well tolerated. The improvements in headache outcomes in this group of patients may be due, in part, to the effect of levetiracetam on electroencephalogram dysrhythmias. These preliminary results of levetiracetam seem to be promising in relieving headaches in the pediatric age group.

No conflict of interest.

